# Differential Activity of Nivolumab, Pembrolizumab and MPDL3280A according to the Tumor Expression of Programmed Death-Ligand-1 (PD-L1): Sensitivity Analysis of Trials in Melanoma, Lung and Genitourinary Cancers

**DOI:** 10.1371/journal.pone.0130142

**Published:** 2015-06-18

**Authors:** Luisa Carbognin, Sara Pilotto, Michele Milella, Vanja Vaccaro, Matteo Brunelli, Anna Caliò, Federica Cuppone, Isabella Sperduti, Diana Giannarelli, Marco Chilosi, Vincenzo Bronte, Aldo Scarpa, Emilio Bria, Giampaolo Tortora

**Affiliations:** 1 Medical Oncology, University of Verona, Azienda Ospedaliera Universitaria Integrata, Verona, Italy; 2 Medical Oncology, Regina Elena National Cancer Institute, Roma, Italy; 3 Department of Pathology and Diagnostic, University of Verona, Azienda Ospedaliera Universitaria Integrata, Verona, Italy; 4 Agenzia Italiana del Farmaco (AIFA), Roma, Italy; 5 Biostatistics, Regina Elena National Cancer Institute, Roma, Italy; 6 ARC-NET Center for Applied Research on Cancer, Verona, Italy; University Campus Bio-Medico, ITALY

## Abstract

**Background:**

The potential predictive role of programmed death-ligand-1 (PD-L1) expression on tumor cells in the context of solid tumor treated with checkpoint inhibitors targeting the PD-1 pathway represents an issue for clinical research.

**Methods:**

Overall response rate (ORR) was extracted from phase I-III trials investigating nivolumab, pembrolizumab and MPDL3280A for advanced melanoma, non-small cell lung cancer (NSCLC) and genitourinary cancer, and cumulated by adopting a fixed and random-effect model with 95% confidence interval (CI). Interaction test according to tumor PD-L1 was accomplished. A sensitivity analysis according to adopted drug, tumor type, PD-L1 cut-off and treatment line was performed.

**Results:**

Twenty trials (1,475 patients) were identified. A significant interaction (*p<0*.*0001*) according to tumor PD-L1 expression was found in the overall sample with an ORR of 34.1% (95% CI 27.6-41.3%) in the PD-L1 positive and 19.9% (95% CI 15.4-25.3%) in the PD-L1 negative population. ORR was significantly higher in PD-L1 positive in comparison to PD-L1 negative patients for nivolumab and pembrolizumab, with an absolute difference of 16.4% and 19.5%, respectively. A significant difference in activity of 22.8% and 8.7% according to PD-L1 was found for melanoma and NSCLC, respectively, with no significant difference for genitourinary cancer.

**Conclusion:**

Overall, the three antibodies provide a significant differential effect in terms of activity according to PD-L1 expression on tumor cells. The predictive value of PD-L1 on tumor cells seems to be more robust for anti-PD-1 antibody (nivolumab and pembrolizumab), and in the context of advanced melanoma and NSCLC.

## Introduction

The concept that the immune system plays a critical role in controlling and eradicating cancer and that immune response, driven by T-lymphocytes, is closely regulated thorough a complicated and delicate balance between inhibitory checkpoints and activating signals is well established [1–4]. Cytotoxic T-lymphocyte antigen 4 (CTLA-4) and programmed death-1 (PD-1) are the two main immune checkpoint receptors that, when binding their ligand B7 and programmed death-ligand-1 (PD-L1) respectively, determine the downregulation of the T-cell effector functions, thus contributing to the maintenance of the tolerance to tumor cells. The blockade of these pathways by the anti-CTLA4, anti-PD-1 and anti-PD-L1 antibodies may prevent this downregulation and allows T-cells to maintain their antitumor property and ability to mediate the tumor cell death [5–7].

With regard to the PD-1 immune-checkpoint pathway, a series of specific inhibitors are currently under investigation and in clinical development such as nivolumab, a fully human IgG4 PD-1 immune checkpoint inhibitor antibody, pembrolizumab (formerly known as MK-3475 or lambrolizumab), a high affinity humanized IgG4 monoclonal antibody targeting PD-1, and MPDL3280A, an engineered IgG anti-PD-L1 antibody [8].

The most promising and revolutionizing data in terms of activity of these immune checkpoint inhibitors derive from advanced melanoma, where pembrolizumab and nivolumab have been recently approved by the U.S. Food and Drug Administration (FDA) for patients with unresectable or advanced melanoma progressing after ipilimumab and a BRAF inhibitor, if BRAF V600 mutation positive [9, 10]. In addition, FDA recently released the fast approval of nivolumab for advanced squamous non-small cell lung cancer (NSCLC) previously treated with platinum-based chemotherapy [11]. Furthermore, encouraging results were also obtained in the context of renal cell (RCC) and bladder carcinoma, and other solid and hematologic disease [12–15].

Overall, the immune checkpoint inhibitors targeting PD-1 and its ligand PD-L1 have shown unprecedented rates of durable clinical responses, with an activity range from 10% to 45% in the context of unselected populations affected by advanced solid tumors [7, 16–19].

In order to identify the proportion of patients most likely to benefit from the immunotherapy and thus to optimize their therapeutic index, the investigation of potential predictive biomarkers represents a relevant aspect of the more recent clinical research.

In this regard, the investigation of the PD-L1 assay (which can be constitutively or inducible expressed on either the surface of tumor cells or upon cancer-infiltrating T-cells) with different methods as a potential biomarker, represents one of the most challenging strategy [20]. PD-L1 is expressed in several types of malignancies and it seems to be associated with worse prognosis in RCC and NSCLC, while with good prognosis in melanoma [21–23].

With respect to the predictive value of the tumor PD-L1 expression, explored in the majority of cases by immunohistochemistry (IHC), it emerged as a potential predictor of response to immune checkpoint inhibitors in a series of clinical trials: one of the first phase I study which enrolled patients with advanced melanoma, NSCLC, castration resistant prostate cancer, RCC and colorectal cancer to receive nivolumab, demonstrated an objective response rate (36%) exclusively in patients with PD-L1 positive tumors [7].

Nevertheless, this finding did not constantly emerge in all studies, thus allowing to speculate about the reliance and reproducibility of the different methods adopted for the detection and the quantification of the biomarker. Indeed, clinical activity was demonstrated in patients with PD-L1 negative tumors [14, 24]. On the basis of these controversial results, the potential predictive role of PD-L1 expression on tumor cells still represents an issue for clinical research.

In order to explore the potential differential activity of nivolumab, pembrolizumab (targeting PD-1) and MPDL3280A (targeting PD-L1) according to the PD-L1 expression on cancer cells in melanoma, NSCLC and genitourinary cancers we conducted a sensitivity analysis of phase I, phase II and phase III trials.

## Methods

The analysis was conducted according to 4 pre-specified steps: 1) definition of the outcomes; 2) definition of the trial selection criteria; 3) definition of the search strategy; and 4) detailed description of the statistical methods used [25, 26].

### Outcome definition

The analysis was conducted to determine the differential activity of nivolumab, pembrolizumab and MPDL3280A in terms of overall response rate (ORR), according to tumor PD-L1 expression for the treatment of advanced melanoma, NSCLC and genitourinary cancers. In presence of significant differential activity, a sensitivity analysis was subsequently performed in the overall sample and according to the following factors: 1) the adopted drug (nivolumab, pembrolizumab and MPDL3280A); 2) the tumor type (melanoma, NSCLC, genitourinary); 3) the PD-L1 expression cut-off (1% and 5%); 4) the treatment line (first line treatment [1°], second and subsequent lines [≥2°], and mixed lines therapy).

### Trial Identification Criteria

All phase I, phase II and phase III clinical trials published in peer-reviewed journals or presented at the American Society of Clinical Oncology (ASCO), the European Society for Medical Oncology (ESMO), the World Conference of Lung Cancer (WCLC), and the Chicago Multidisciplinary Symposium in Thoracic Oncology (CMSTO) meetings up to February 15^th^, 2015 in which patients affected by advanced melanoma, NSCLC and genitourinary cancers (RCC and bladder carcinoma) were assigned to receive anti-PD-1/PD-L1 antibody therapy including nivolumab, pembrolizumab and MPDL3280A as single agent or in combination with other immunotherapies such as vaccines or checkpoint antibodies were considered. As inclusion criteria, eligible arms for the current analysis had to report the ORR, assessed by the Response Evaluation Criteria in Solid Tumors (RECIST), according to PD-L1 expression at IHC evaluated in cancer cells with the cut-off more frequently represented in the selected trials (1% and 5%). Other antibodies targeting the PD-1/PD-L1 axis in less advanced stage of clinical development, such as those including pidilizumab, MDX-1105, MEDI4736, MSB0010718C, AMP-224 were excluded. Studies employing nivolumab, pembrolizumab or MDPL3280A in combination with target therapies or chemotherapic agents and trials with PD-L1 expression evaluated in tumor infiltrating immune cells, or different assay from IHC, were excluded as well.

### Search Strategy

Deadline for trial publication and/or presentation was February 15^th^, 2015. Updates of trials were gathered through Medline (Pubmed: www.ncbi.nlm.nih.gov/PubMed), ASCO (www.asco.org), ESMO (www.esmo.org), WCLC (www.iaslc.org), and CMSTO (www.thoracicsymposium.org) searches. Keywords used for searching were: PD-L1, PD-1, nivolumab, pembrolizumab, MPDL3280A, melanoma, NSCLC, bladder carcinoma, RCC, phase I, phase II, phase III. In addition to computer browsing, review and original papers were also scanned in the references section to look for missing trials. Furthermore, lectures at major meetings having immune checkpoint blockade for advanced solid tumor’ as the topic were checked.

### Data Extraction

Data for selected outcomes were extracted: the last available update of each trial was considered as the original sources. Data for each explored outcome were extracted from trials according to the following criteria: 1) adopted drug; 2) tumor type; 3) PD-L1 expression cut-off; 4) treatment line. All data were reviewed by 3 investigators (L.C., S.P., E.B.) and separately computed by 4 investigators (L.C., I.S., D.G. and E.B.) [26].

### Data Synthesis

Overall response rate according to RECIST were extracted from papers and/or presentations and 95% confidence intervals (CIs) were derived [27–29]. Data were cumulated by adopting a fixed and random-effect model according to the Der Simonian and Laird method [27].

The analysis to test for interaction (Cochrane-Q) according to the PD-L1 categorical expression (positive versus negative, as defined by trialists) was accomplished. The sensitivity analysis was conducted in the context of: 1) the adopted drug (nivolumab, pembrolizumab and MPDL3280A); 2) the tumor type (melanoma, NSCLC and genitourinary); 3) the PD-L1 expression cut-off (1% and 5%); 4) the treatment line (1°, ≥2° and mixed) [30, 31]. In presence of significant results, the chi-square test was adopted to determine differences between rates.

Calculations were accomplished using the licensed Comprehensive Meta-analysis (version 2.0, CMA, biostat, Englewood, NJ, USA), and MedCalc (version 14.12.0, MedCalc Software bvba, Ostend, Belgium) software.

## Results

### Selected trials

**Fig 1:**
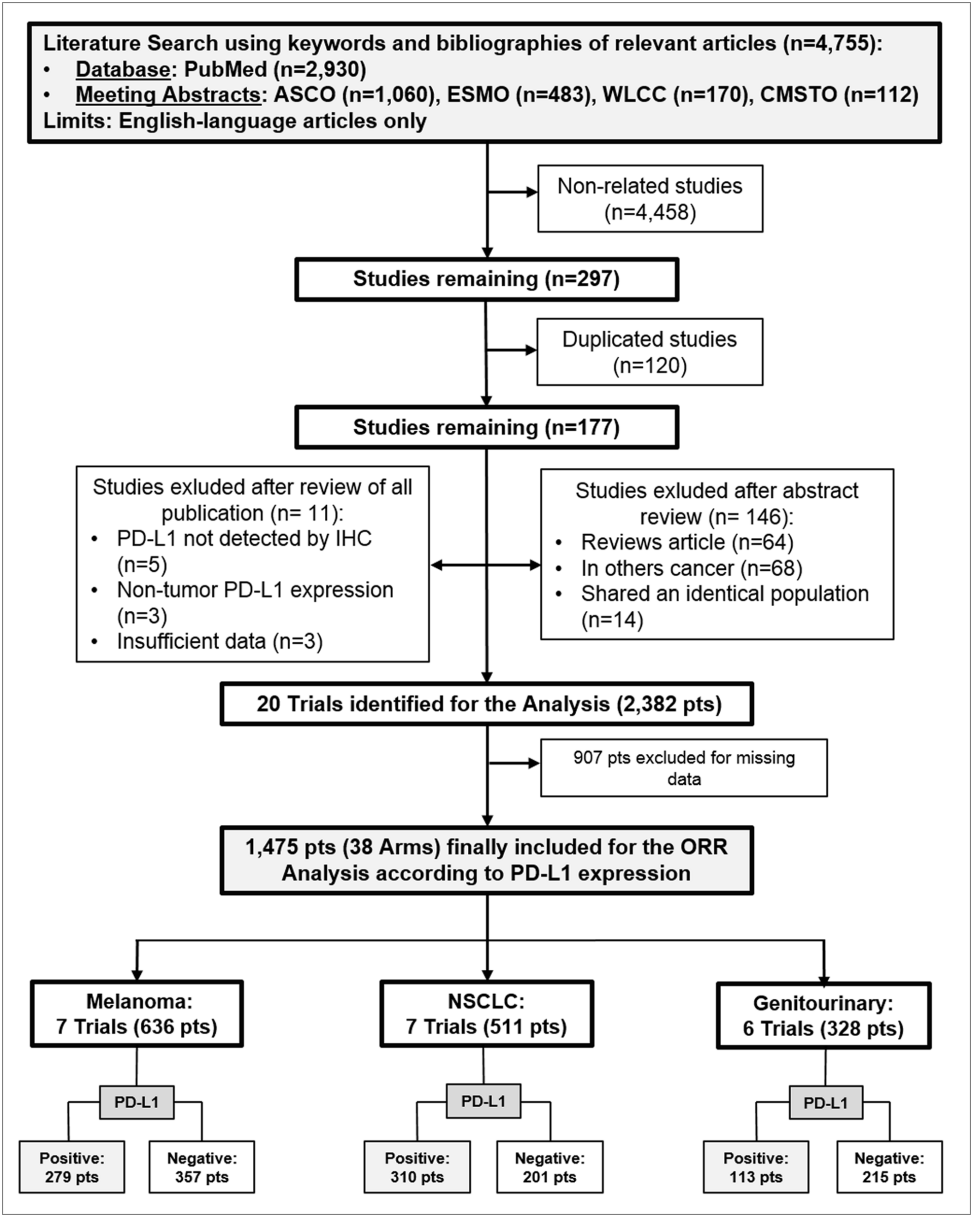
Outline of the search—Flow chart of the studies included in the analysis. Pts: patients; n: number; PD-L1: programmed death-ligand-1; IHC: immunohistochemistry; ORR: overall response rate; NSCLC: non-small cell lung cancer.

Twenty trials (2,382 patients) were identified [10, 11, 14, 15, 24, 32–46]. Data from 38 arms (1,475 patients) of the original 20 trials were considered for the analysis (Fig 1). Of these, 12 trials (921 patients) [10, 11, 14, 24, 32, 35, 38–40, 43–45] incorporated a nivolumab-based, 4 trials (382 patients) [36, 37, 41, 46] a pembrolizumab-based and 4 trials (172 patients) [15, 33, 34, 42] a MPDL3280A-based treatment. Trials characteristics and selected arms for the analysis are listed in Table 1.

**Table 1:** Trials’ Characteristics (selected arms for the analysis).

Author	Phase	Disease Type	Drug	Treatment Line	PD-L1Cut-Off (%)	PD-L1 Subgroup	Sample size	ORR(%)
***Weber et al, JCO 2013 [39]***	I	Melanoma	Nivolumab	≥2°	5	Positive	12	**67.0**
						Negative	32	**19.0**
***Hamid et al, ASCO 2013 [33]***	I	Melanoma	MPDL3280A	Mixed	5	Positive	15	**27.0**
						Negative	15	**20.0**
***Hodi et al, ASCO 2014 [35]***	I	Melanoma	Nivolumab	≥2°	5	Positive	18	**44.0**
						Negative	23	**13.0**
***Sznol et al, ASCO 2014 [38]***	I	Melanoma	Nivolumab	≥2°	5	Positive	22	**35.0**
						Negative	57	**59.0**
***Robert et al, NEJM 2015 [24]***	III	Melanoma	Nivolumab	1°	5	Positive	74	**52.7**
						Negative	136	**33.1**
***Weber et al, LO 2015 [10]***	III	Melanoma	Nivolumab	≥2°	5	Positive	55	**43.6**
						Negative	64	**20.3**
***Kefford et al, ASCO 2014 [36]***	I	Melanoma	Pembrolizumab	Mixed	1	Positive	83	**49.0**
						Negative	30	**13.0**
***Rizvi et al, CMSTO 2014 [43]***	I	NSCLC	Nivolumab	1°	5	Positive	26	**31.0**
						Negative	21	**10.0**
***Antonia et al, CMSTO 2014 [44]***	I	NSCLC	Nivolumab	1°	5	Positive	16	**19.0**
						Negative	22	**14.0**
***Herbst et al, Nature 2014 [34]***	I	NSCLC	MPDL3280A	≥2°	5	Positive	9	**27.0**
						Negative	37	**24.0**
***Gettinger et al, JCO 2015 [45]***	I	NSCLC	Nivolumab	≥2°	5	Positive	33	**15.0**
						Negative	35	**14.0**
***Rizvi et al, LO 2015 [11]***	II	NSCLC	Nivolumab	≥2°	5	Positive	25	**24.0**
						Negative	51	**14.0**
***Garon et al, NEJM 2015 [46]***	I	NSCLC	Pembrolizumab	≥2°	1	Positive	159	**23.0**
						Negative	35	**9.0**
***Rizvi et al, ASCO 2014 [37]***	I	NSCLC	Pembrolizumab	1°	1	Positive	42	**26.0**
***Cho et al, ASCO 2013 [42]***	I	GU	MPDL3280A	Mixed	5	Positive	10	**20.0**
						Negative	21	**10.0**
***Powles et al, Nature 2014 [15]***	I	GU	MPDL3280A	≥2°	5	Positive	7	**29.0**
						Negative	58	**26.0**
***Motzer et al, JCO 2014 [14]***	II	GU	Nivolumab	≥2°	5	Positive	29	**31.0**
						Negative	78	**18.0**
***Choueiri et al, ESMO 2014 [32]***	I	GU	Nivolumab	Mixed	5	Positive	18	**22.0**
						Negative	38	**8.0**
***Hammers et al, ESMO 2014 [40]***	I	GU	Nivolumab	Mixed	1	Positive	16	**50.0**
						Negative	20	**55.0**
***Plimack et al, ESMO 2014 [41]***	I	GU	Pembrolizumab	Mixed	1	Positive	33	**24.1**

PD-L1: programmed death-ligand-1; ORR: overall response rate; NSCLC: non-small cell lung cancer.

With regard to tumor type, 7 studies (636 patients) [10, 24, 33, 35, 36, 38, 39] included melanoma, 7 studies (511 patients) [11, 34, 37, 43–46] included NSCLC, and 6 studies (328) [14, 15, 32, 40–42] included genitourinary cancer. Considering the PD-L1 cut-off expression, 5 trials (418 patients) [36, 37, 40, 41, 46] reported cut-off of 1% and 15 trials (1057 patients) [10, 11, 14, 15, 24, 32–35, 38, 39, 42–45] reported cut-off of 5%. With respect to treatment line, 4 trials (337 patients) [24, 37, 43, 44] explored first line setting, 10 trials (839 patients) [10, 11, 14, 15, 34, 35, 38, 39, 45, 46] explored second and subsequent lines setting, and 6 trials (299 patients) [32, 33, 36, 40–42] mixed line setting.

### Interaction Analysis

**Fig 2:**
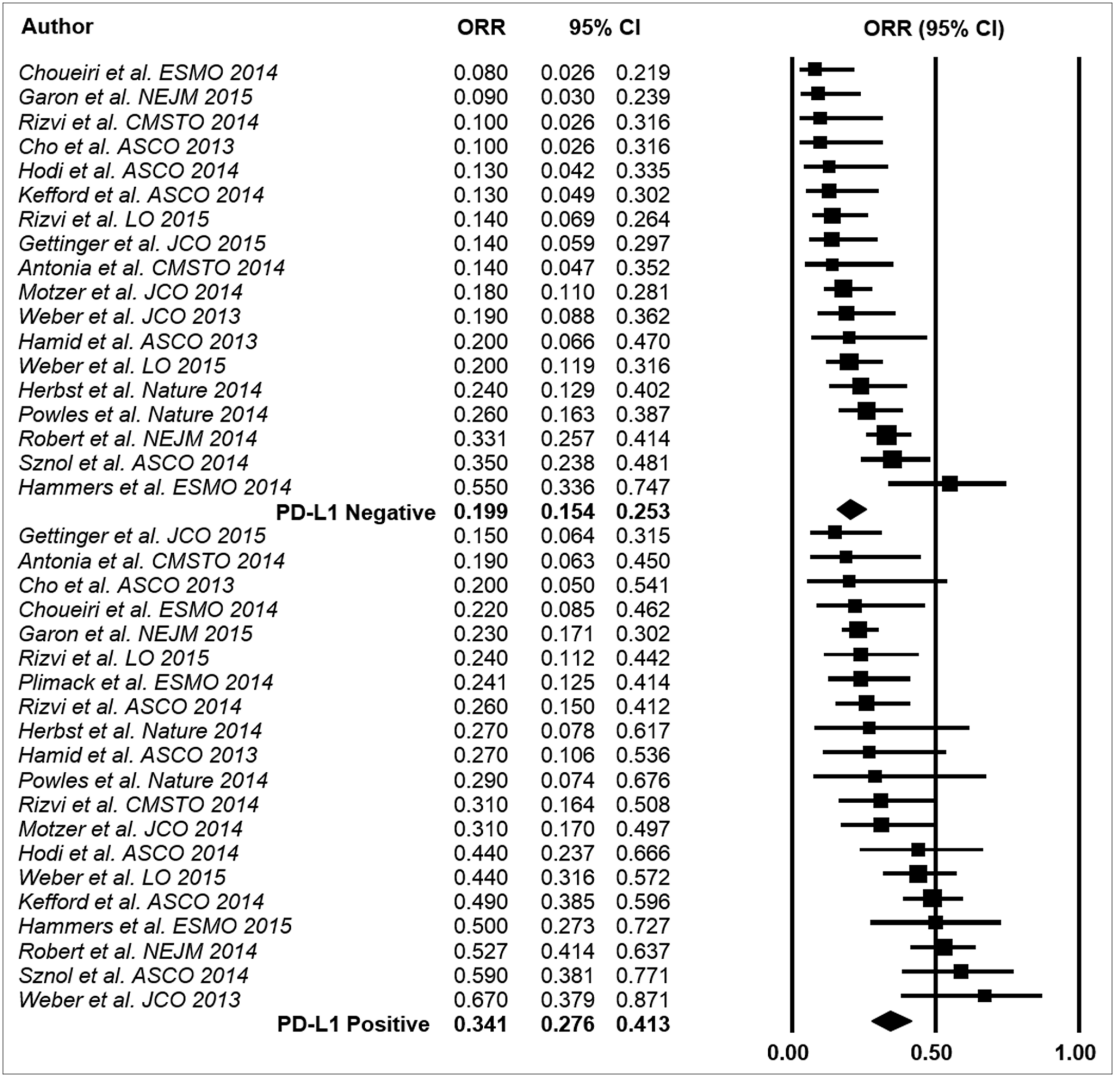
Results of the event rate analysis. ORR: overall response rate; CI: confidence interval; PD-L1: programmed death-ligand-1.

A significant interaction (*p<0*.*0001*) according to tumor PD-L1 expression was found in the overall sample with an ORR of 34.1% (95% CI 27.6–41.3%) in the PD-L1 positive population and 19.9% (95% CI 15.4–25.3%) in the PD-L1 negative population (Fig 2).

### Sensitivity Analysis

**Fig 3:**
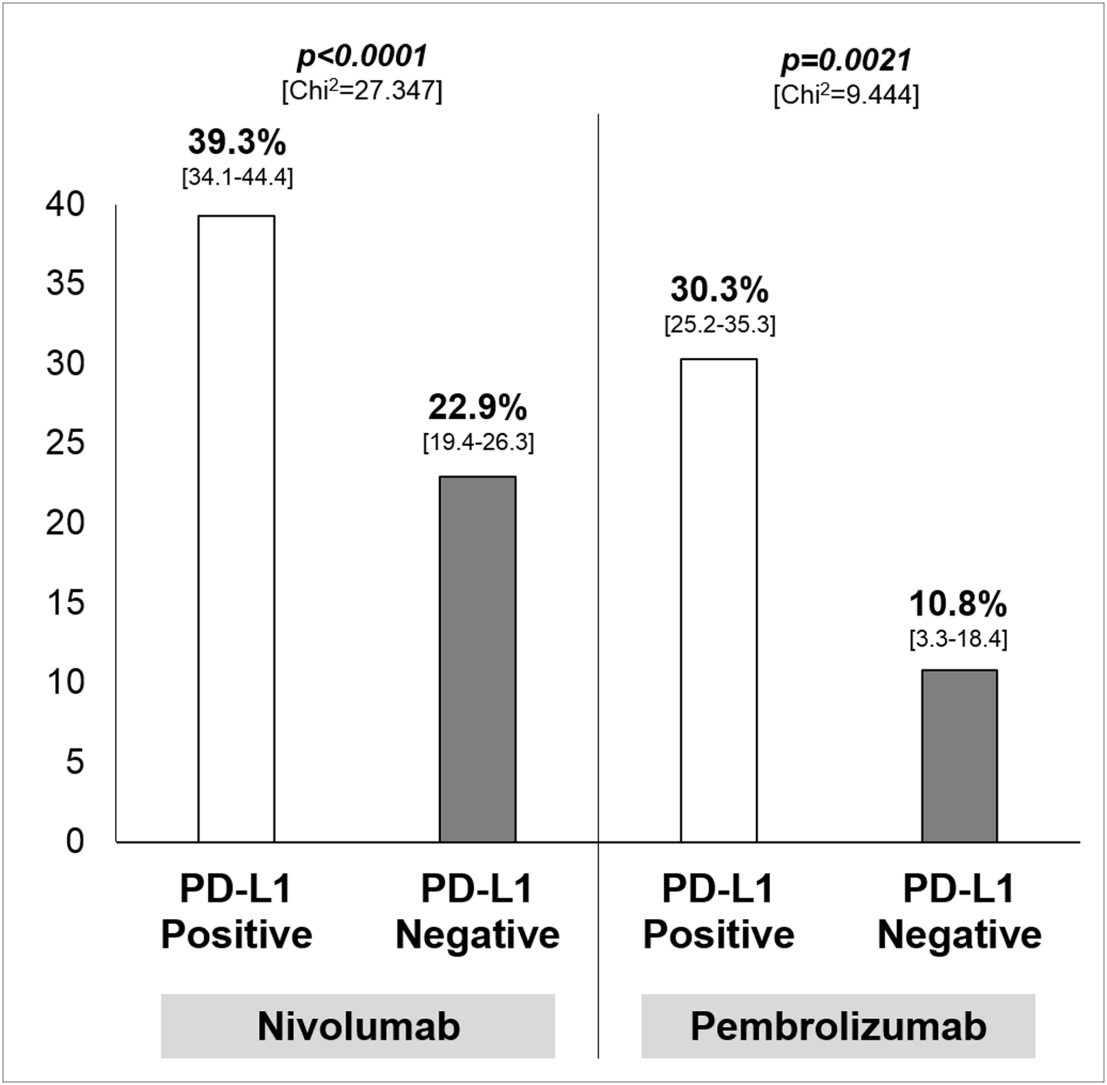
Results of the sensitivity analysis—Overall response rate, with 95% confidence interval in square brackets, according to adopted drug. Chi^2^: Chi-square test; PD-L1: programmed death-ligand-1.

With regard to the adopted drug, ORR was significantly higher in patients with PD-L1 positive tumor in comparison to PD-L1 negative tumor for nivolumab and pembrolizumab, with an absolute difference of 16.4% (95% CI 10.0–22.7) and 19.5% (95% CI 8.1–27.8) respectively (Fig 3). Results with regard to MPDL3280A are not reported given the small number of treated patients; nevertheless, no significant difference according to PD-L1 expression was found for this drug (*p = 0*.*809*, data not shown).

**Fig 4:**
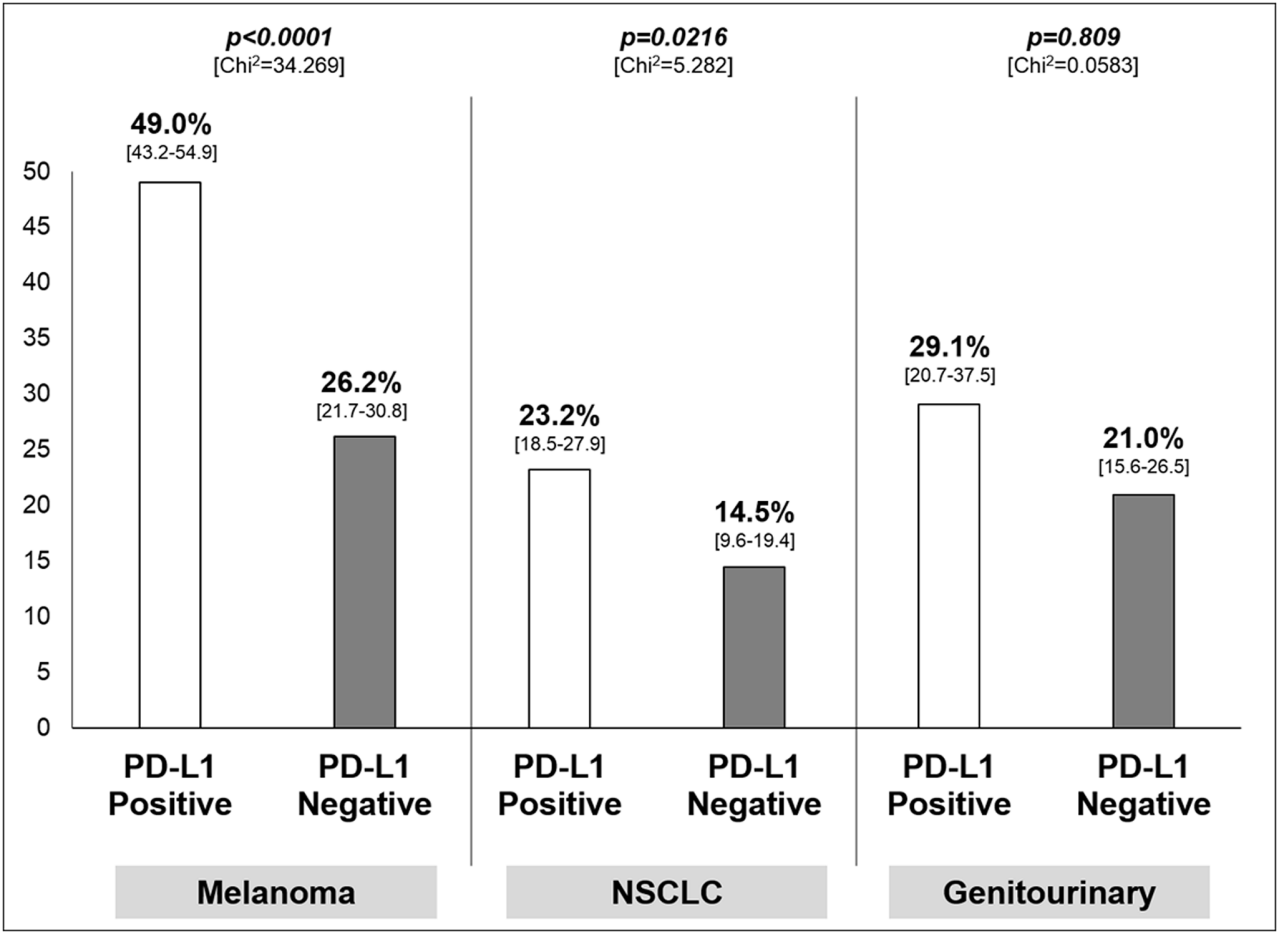
Results of the sensitivity analysis—Overall response rate, with 95% confidence interval in square brackets, according to tumor type. Chi^2^: Chi-square test; PD-L1: programmed death-ligand-1; NSCLC: non-small cell lung cancer.

For what concerns the tumor type, patients with PD-L1 positive tumor significantly achieved more responses than PD-L1 negative, with an absolute difference of 22.8% for melanoma (95% CI 12.5–30.3) and 8.7% for NSCLC (95% CI 1.1–15.5) as shown in Fig 4. With respect to genitourinary cancer, the difference was not significant (*p = 0*.*809*).

**Fig 5:**
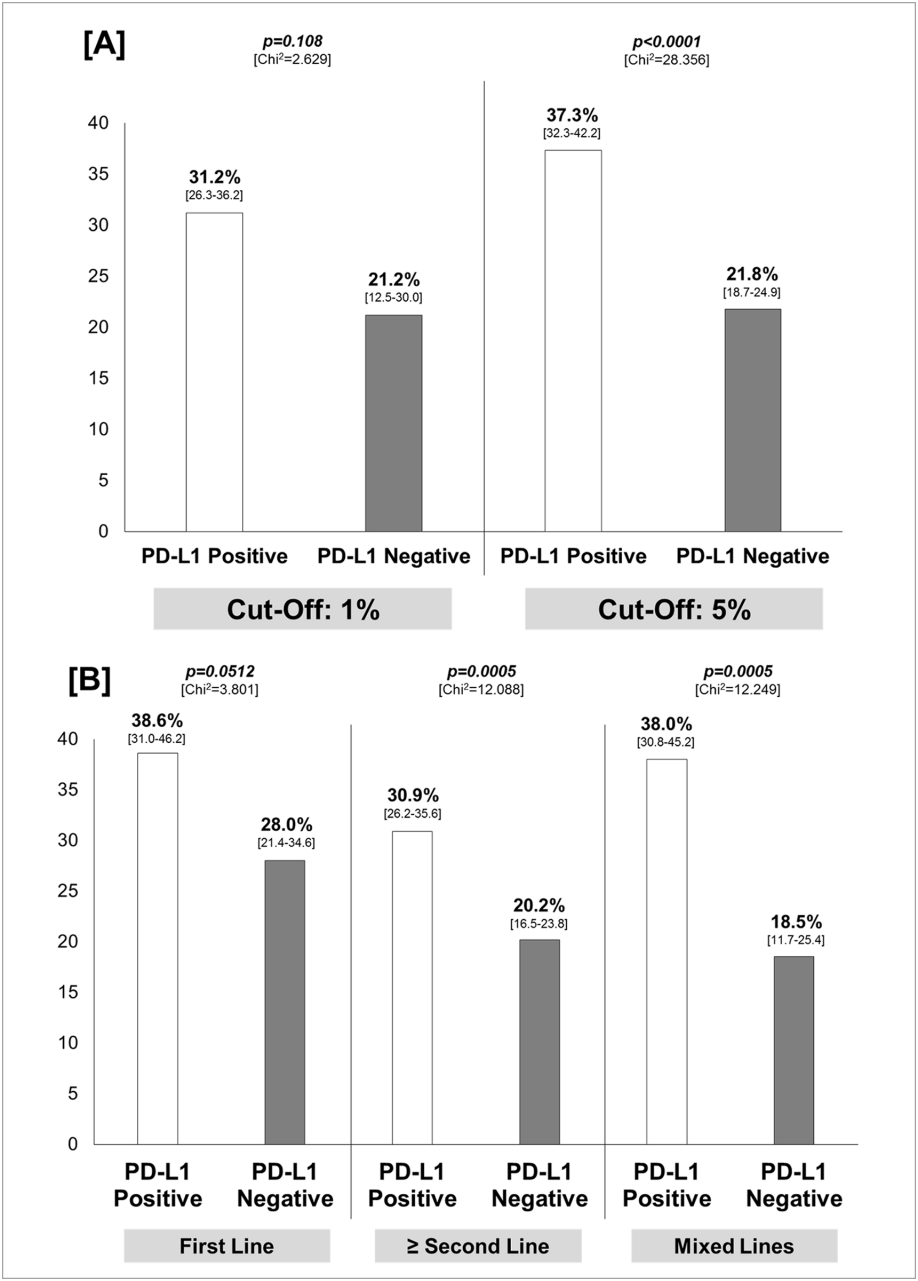
Results of the sensitivity analysis. *Panel [A]*: overall response rate, with 95% confidence interval in square brackets, according to PD-L1 expression cut-off; *Panel [B]*: overall response rate with 95% confidence interval in square brackets, according to treatment line. Chi^2^: Chi-square test; PD-L1: programmed death-ligand-1.

Considering the PD-L1 expression cut-off, the difference in ORR between PD-L1 positive and negative patients was significantly higher in studies adopting the cut-off of 5%, with an absolute difference of 15.5% (95% CI 9.5–21.4). No significant difference is seen for trials where the cut-off was 1% (*p = 0*.*108*) (Fig 5, Panel A).

In the context of first line treatment, a trends towards significance for patients with PD-L1 positive tumor was found (*p = 0*.*0512*); conversely, the difference was statistically significant in the context of second or subsequent and mixed lines with an absolute difference of 10.7% (95% CI 4.6–16.8) and 19.5% (95% CI 8.7–29.4), respectively (Fig 5, Panel B).

## Discussion

According to the results reported herein, the checkpoint inhibitors targeting PD-1 (nivolumab and pembrolizumab) and its ligand PD-L1 (MPDL3280A) provide a significant differential effect in terms of activity according to PD-L1 expression on tumor cells status, which translates into a higher ORR for PD-L1 positive advanced tumors in comparison to PD-L1 negative tumors, with an overall absolute difference of 14.2% (Fig 2).

The secondary objective of the analysis was to evaluate if the differential effect remained robust even in the context of the different adopted drugs, tumor types, PD-L1 cut-off expressions and treatment lines.

With regard to the sensitivity analysis according to the adopted drug, PD-L1 status seems to predict the chance of response for nivolumab and pembrolizumab (Fig 3), while data referring to the activity of MPDL3280A indicating no differences according to PD-L1 should be considered too early. If that depends from the different mechanism of action and the different targets of the 3 antibodies (nivolumab and pembrolizumab against PD-1, MPDL3280A against PD-L1) represents a matter of research. In this regard, our data provide a similar difference in terms of ORR difference between PD-L1 positive and PD-L1 negative tumor for nivolumab and pembrolizumab (16–19%, Fig 3). Nevertheless, a formal comparison between drugs should not be performed given the strong difference in terms of population size: indeed, the overall number of patients treated with nivolumab, pembrolizumab and MPDL3280A was 921, 382, and 172, respectively, with only 41 patients PD-L1 positive receiving MPDL3280A.

Whit respect to disease setting, our analysis shows that a significant higher ORR in PD-L1 positive tumor than PD-L1 negative tumor is documented for melanoma and NSCLC while a significant effect is not documented in the context of genitourinary cancer, despite an absolute difference of 8.1%. Across the tumor types, although we cannot perform a comparison between the different disease settings, the most overall ‘immuno-sensitive’ disease seems to be melanoma (as expected) where, even in the worst case (PD-L1 negative tumors) an activity of 26.2% is documented (Fig 4).

The PD-L1 IHC positivity on tumor cells was defined by a 1% or 5% threshold in most of the selected studies. In these studies, besides the various cut-off, various methodologies were adopted in the context of IHC such as different anti-PD-L1 antibodies, staining techniques (manual or automated assay), the definitions of ‘positive’ tumor (cell surface versus cytoplasmic expression), the definitions of PD-L1 ‘positive’ patients (based on a single tumor biopsy, or on maximal expression in the case of multiple biopsies from an individual patient) and the sample used for the assay (primary tumor or metastatic lesion).

In the more recent release of the KEYNOTE-001 trial, which evaluated pembrolizumab in advanced NSCLC, a cut off of 50% was adopted to define the IHC PD-L1 positivity: patients were divided into three groups, based on whether they had membranous PD-L1 expression in their tumor cells of ≥50%, 1%-49%, or <1%. Patients’ survival significantly differed between patients with high PD-L1 (1-year OS >50%) in comparison with those patients with a PD-L1 lower than 50%, who did not overcome a 1-year OS of nearly 28% [46].

The use of multiple proprietary PD-L1 IHC assays, whose comparative outcomes are not well established, and the lack of a clear definition of ‘positive’ tumor-PD-L1 represent a limit for the interpretation of data from clinical trials according to biomarkers [47].

According to our results, despite the heterogeneity, the best cut-off able to discriminate between ‘responders’ and ‘not responders’ seems to be 5% (Fig 5, Panel A). With regard to PD-L1 assessment, the analytical validation of an easy and reproducible test, as the IHC assay, that may be successfully and clinically useful to best discriminate sub-population most likely to benefit from treatment with immune check point inhibitors represents a key question.

In addition, patients with PD-L1 positive tumor had a significantly higher chance to respond to treatment regardless of the treatment line (Fig 5, Panel B).

Besides the heterogeneity of the PD-L1 expression assessment, another limitation of the analysis is the heterogeneity of the included studies (few phase II with ORR as primary objective were available). The hypothesis that a differential effect according to the biomarker analyzed exist seems to be reasonable regardless of the anti-PD-1 drugs, tumor types (melanoma and NSCLC), and treatment lines. With this promise, how much this differential activity in terms of response according to the tumor PD-L1 expression status translates into a significant outcome benefit for the patients? This questions represents an interest and recent matter of research.

On the basis of the available phase III trials, the KEYNOTE-066 study, demonstrated an overall survival benefit with pembrolizumab in patients with advanced melanoma, as compared to ipilimumab, across all subgroups except for the small subgroup of patients (less than 20%) with PD-L1 negative tumors [48].

The CheckMate 066 study conducted by *Robert et al* to determine whether nivolumab, as compared with dacarbazine, improves overall survival among previously untreated advanced melanoma patients, demonstrated a survival benefit with nivolumab regardless of tumor cells PD-L1 expression status. However, these results are still preliminary as in the nivolumab group the median overall survival was not reached in either PD-L1 subgroup. Interestingly, in the dacarbazine group, the median overall survival was slightly longer in the subgroup with positive PD-L1 status than in the subgroup with negative or indeterminate PD-L1 status (12.4 vs. 10.2 months), thus opening the possible role of PD-L1 as prognostic factors, which actually still remains to be determined. Overall, the authors concluded that, given the magnitude of the clinical benefit observed in patients receiving nivolumab, PD-L1 status alone, does not seem to be useful in the selection of patients for nivolumab treatment [24].

In this regard, the reason why even patients with PD-L1 negative tumor respond and why the majority of patients with PD-L1 positive tumor do not response to PD-1 pathway blockade represents an area of ongoing research. Recent studies demonstrate that besides the PD-L1 expression by tumor cells, the expression of PD-L1 on immune cells infiltrating the tumor is a potential predictor of clinical response [49].

Furthermore, in the study of *Herbst et al* the association of tumor infiltrating immune cell PD-L1 expression with treatment response to MPDL3280A in several solid tumor types appears stronger than that with tumor cell PD-L1 expression [34]. Similar results are reported in the adaptive design trial conducted by *Powels et al* in the context of metastatic bladder cancer treated with MPDL3280A [15]. Conversely, an analysis of multiple factors in pretreatment tumor specimens from patients with advanced cancers receiving anti—PD-1 (nivolumab) therapy demonstrated that only the tumor cell PD-L1 expression is most closely associated with objective tumor regression; the other micro-environmental features analyzed, such as tumor infiltrating lymphocytes PD-1 expression and the intensity of T-cell and B-cell infiltrates, are associated with PD-L1 expression on tumor or tumor infiltrating immune-cells, but not independently associated with treatment response [50].

Overall, these results are in agreement with our sensitivity analysis data, where the predictive value of PD-L1 on tumor cells seems to be consistent just for anti-PD-1 antibody. Despite still unclear, several other mechanisms and immune regulatory pathways seem to be involved in the response to PD-1/PD-L1 pathway blockade such as the PD-L2 expression, a second known ligand for PD-1, the PD-1 expression on T-lymphocytes, and the discovery of immunogenic neo-antigens, encoded by gene mutations called ‘passenger’ that do not trigger the cancer development but play an important role in immunogenicity [34, 51–53].

In this regard, even the results reported by *Snyder* and colleagues in the context of advanced melanoma treated with CTLA-4 blockade demonstrated that a high mutational burden providing a greater likelihood of the development of specific tumor neo-antigens, recognized by the T-cells, is associated with a long-term clinical benefit from CTLA-4 blockade; conversely the absence of mutation-derived neo-antigens is associated with a minimal benefit or no benefit [54, 55]. Very same data were recently reported for NSCLC patients treated with pembrolizumab [56]. Another aspect is that the immune system may be dynamic; thus, the evaluation of a potential biomarker at a single time point (for example baseline) may not reflect an evolving immune response in the tumor microenvironment [49].

Despite the overall heterogeneity, the non-prospective comparison according to PD-L1, and the fact that ORR according to this biomarker was not determined in all treated patients, the results reported herein show that patients affected by melanoma, NSCLC and genitourinary with positive PD-L1 on tumor cells may have a higher chance of response to nivolumab, pembrolizumab, and (with limited confidence) MPDL3280A in comparison to PD-L1 negative tumor. Besides the pure significant statistical effect, the magnitude of the benefit may be clinically significant in some clinical context as well. For example, with respect to the second or subsequent line treatment for metastatic NSCLC, where the maximum activity of the chemotherapy is around 10–15%, the identification of a predictive biomarker able to improve ORR over 20% in the PD-L1 positive tumor should be considered clinically meaningful [11]. The predictive role of PD-L1 tumor expression which appear to be consistent across drugs and diseases, may be not of value when anti-PD-1/PD-L1 immune checkpoint inhibitors are administered concurrently with ipilimumab, where ORR did not differ according to PD-L1 [40, 57].

In conclusion, the PD-L1 expression may potentially represent a reasonable candidate biomarker for the patients’ selection in order to optimize the treatment strategy with immune checkpoint antibodies. Nevertheless, the analytical validation and standardized definition of PD-L1 expression on tumor cells or infiltrating immune cells according to a shared cut-off to define positivity are warranted. In addition, the clinical validation of this potential biomarker as a predictor of response requires further phase III trials stratified according to tumor PD-L1 status and prospective studies in large cohorts of patients with PD-L1 positive or negative disease. Clinical trials aiming to understand the complex tumor immune microenvironment and to identify additional predictive markers or gene signature of response or resistance to immunotherapy, are currently needed.

## Supporting Information

S1 Checklist PRISMA checklist. (PDF)
